# Mesenchymal stem cell-derived exosomes can alleviate GVHD and preserve the GVL effect in allogeneic stem cell transplantation animal models

**DOI:** 10.3389/fimmu.2023.1284936

**Published:** 2023-12-06

**Authors:** Yan Jiang, Jie Zhao, Minghui Wang, Fang Huang, Jiaqi Li, Rui Liu, Jiangbo Wan, Siguo Hao

**Affiliations:** Department of Hematology, Xinhua Hospital Affiliated to Shanghai Jiao Tong University School of Medicine, Shanghai, China

**Keywords:** mesenchymal stem cells, exosomes, hematopoietic stem cell transplantation, graft-versus-host disease, graft-versus-leukemia

## Abstract

**Background:**

Mesenchymal stem cells (MSCs) can alleviate graft-versus-host disease (GVHD) in hematopoietic stem cell transplantation (HSCT). MSCs-derived exosomes (MEXs) can mirror the biological function of their parent cells. Whether MEXs can alleviate GVHD like their parent cells or not is unclear. In this study, we investigate the effects of MEXs on GVHD and graft-versus-leukemia (GVL) effect *in vitro* and in HSCT animal models.

**Method:**

MSCs were produced using bone marrow mononuclear cells (MNCs), and MEXs were separated from the supernatants of MSCs. Electron microscopy, western blot, and nanoparticle tracking analysis (NTA) were used to determine the characteristics of MEXs. The immunomodulatory function of MEXs and their effects on GVHD and GVL were examined *in vitro* and *in vivo*.

**Result:**

Like other cell-type derived exosomes, our data revealed that MEXs were also disc-shaped vesicles with a diameter of 100–200 nm under electron microscopy and were positive for the exosomal hallmark proteins. MEXs can notably inhibit the expression of costimulatory molecules and functional cytokine secretion of dendritic cells (DCs). Meanwhile, MEXs can exert suppressive effects on T lymphocyte proliferation and activation. Moreover, MEXs can also encourage the polarization of macrophages toward the M2 type. In animal HSCT models, MEXs can promote the differentiation of Treg cells in spleens, decrease the GVHD score, increase the survival rate of mice, and preserve the cytotoxic antileukemia effects of CD8^+^ T lymphocytes from recipient mice.

**Conclusion:**

These findings showed that MEXs exert their effects by inhibiting the immunomodulatory function of DCs, macrophages, and T lymphocytes. In the animal model, MEXs ameliorate the clinical symptoms of GVHD, while maintaining the antitumor effects of CD8^+^ T lymphocytes. Therefore, it can be inferred that MEXs can separate GVHD from GVL in HSCT. Our study suggests that MEXs have broad clinical application potential in the prevention and treatment of GVHD in HSCT in the near future.

## 1 Introduction

Graft-versus-host disease (GVHD) is a frequent complication of allogeneic hematopoietic stem cell transplantation (HSCT) and is also one of the most important causes of death of HSCT recipients. The allogeneic T lymphocytes from the donor to the recipient are the protagonists that initiate the development of GVHD. On the other hand, donor T cells can also recognize and eradicate residual leukemic cells and induce graft-versus-leukemia (GVL) effect, which reduces the probability of leukemia recurrence after transplantation. Therefore, a crucial factor in raising the success rate of allo-HSCT is reducing GVHD while maintaining the GVL effect.

Mesenchymal stem cells (MSCs) constitute a subset of non-hematopoietic pluripotent stem cells that can differentiate into osteoblasts, chondrocytes, adipocytes, and other cell types that are frequently used in tissue regeneration and repair. The MSCs can be isolated from bone marrow, umbilical cord, adipose tissue, placenta, and other tissues (1, 2). Their anti-inflammatory and immunomodulatory properties have gained increasing attention in recent years (3–5). It was demonstrated that MSCs prevent T cells from differentiating into helper T cells (Th17 and Th1 cells) (6, 7). Inflammatory conditions enhance the potential of MSCs to recruit inflammatory Th17 cells, induce a T regulatory cell phenotype, and enhance IL-4, IL-5, IL-13, and IL-10 production by differentiating Th17 cells (6). Jung et al. demonstrated that mesenchymal stromal cell-derived extracellular vesicles (MSC-EVs) exert their therapeutic effects by degrading RORγt at the protein level, leading to Th17 cell depolarization through K63-linked deubiquitination as a Th17 cell inhibitor (8).

Moreover, MSCs demonstrated their efficacy in GVHD prevention and treatment in allo-HSCT due to their immunoregulatory function. Dotoli et al. reported that patients with steroid-resistant GVHD were treated with third-party MSCs, and 50% of patients showed clinical improvement, 13% had a complete response, and 60.9% had a partial response (9). A meta-analysis showed that the incidence of grade II-IV acute GVHD and chronic GVHD was significantly lower in patients in the MSCs prophylaxis group compared to the control group without MSCs prophylaxis, and patients had a 17% increase in overall survival (10). In addition to suppressing the immune control of GVHD, MSCs promoted the implantation of hematopoietic stem cells, and it was concluded that MSCs are a component of the hematopoietic microenvironment and act as a promoter of hematopoiesis (11). Therefore, MSCs have clinical application prospects in HSCT.

Exosomes (EXOs), the most prevalent type of extracellular vesicle, are tiny vesicles released from eukaryotic cells when the endosomes fuse with the plasma membrane. EXOs have a diameter of 40–100 nm and contain proteins and complex RNAs of parental origin (12). Previous studies have shown that, like MSCs, MSCs-derived exosomes (MEXs) or mesenchymal stromal cell-derived extracellular vesicles (MSC-Evs) can also exert suppressive immunomodulatory actions on T lymphocytes and antigen-presenting cells (13–15). Additionally, MEXs can inhibit the activation and function of B-lymphocytes while suppressing the secretion of B-cell immunoglobulins (16). Another study showed that MEXs can promote the polarization of macrophages to M2 type cells, which is accompanied by the downregulation of Interferon-γ (IFN-γ), interleukin-12 (IL-12), and tumor necrosis factor-α (TNF-α) expression and the upregulation of the immune-negative regulator IL-10 (17). Taken together, MEXs can mirror the immunomodulatory functions of their parent MSC cells. In addition, in clinics, MEXs may have several advantages over MSCs, including cell-free nature, mass production, easy preservation, and transportation (18). Therefore, in this study, we will determine whether MEXs, like their parent MSC cells, have comparable therapeutics on GVHD, and examine their effects on GVL *in vitro* and in animal HSCT models.

## 2 Method

### 2.1 Materials and reagents

Fetal bovine serum (FBS), Dyna beads Mouse T-Activator CD3 and CD28 beads, penicillin, and streptomycin were purchased from Gibco BRL/Life Technologies (Grand Island, NY, USA). IL-2 (212–12–5), GM-CSF (315-03-20), and IL-4 (214-14-20) were purchased from PeproTech (Rocky Hill, NJ, USA). Exosome-depleted FBS was obtained from System Biosciences (Mountain View, CA, USA). CFSE and LDH cytotoxicity assay kits were obtained from Invitrogen (Shanghai, China). Rabbit anti-mouse TSG101 (ab125011), CD63 (ab217345), and CD9 (ab92726) were purchased from Abcam (Shanghai, China). The anti-GAPDH (GB11002) was bought from Servicebio (Wuhan, Hubei, China). Anti-mouse CD29-APC (17-0291-80), anti-mouse CD8a-APC (17-0081-81), anti-mouse CD69-FITC (11-0691-82), anti-mouse Foxp3-APC (17-5773-82), anti-human/mouse Granzyme B-PE (372208), anti-mouse CD8a-FITC (100706), anti-mouse Perforin-APC (154404), and Foxp3/transcription factor staining buffer set were acquired from eBioscience (San Diego, CA, USA) for flow cytometry analysis. Anti-mouse CD11c-PE (117307), anti-mouse CD45R/B220-PE (103207), anti-mouse Sca1-PE (108107), anti-mouse CD80-APC (104713), anti-mouse CD86-APC (105011), anti-mouse CD40-APC (124611), anti-mouse/human CD11b-FITC (101205), anti-mouse F4/80-APC (23115), anti-mouse CD206-PE (141705), anti-mouse H-2K^b^-APC (116517), and anti-mouse H-2K^d^-PE (116607) were obtained from Biolegend (San Diego, CA, USA). The cytometric bead array (CBA) mouse Th1/Th2 cytokine kit was purchased from BD Biosciences (San Diego, CA, USA).

### 2.2 Cell lines and animals

The A20 cell line, a B-cell lymphoblastic leukemia cell line of BALB/c origin, and the RAW264.7 cell line, a macrophage cell line, were obtained from the American Type Culture Collection (Manassas, VA, USA). Male mice of C57BL/6 (H-2k^b^) and BALB/c (H-2k^d^) strains, aged 8–10 weeks, were purchased from the Shanghai Laboratory Animal Center (SLAC; Shanghai, China). The mice were housed under specific pathogen-free conditions. All animal tests were carried out in accordance with the regulations set forth by the Ethics Committee of Xinhua Hospital Affiliated with the Shanghai Jiao Tong University School of Medicine (Shanghai, China).

### 2.3 Generation and identification of MSCs

C57BL/6 mice were sacrificed, and the bone marrow cells from femurs and tibias were extracted by flushing with a DMEM complete medium. The washing fluid was filtered through a 40-µm strainer, and the cells were collected by centrifugation (1000 rpm, 23 °C, 5 min). The pellet was resuspended in a low-glucose DMEM medium supplemented with 10% FBS, penicillin (100 U/ml), and streptomycin (100 U/ml) and incubated at 37°C in a humidified atmosphere containing 5% CO_2_. To remove the non-adherent T lymphocytes, a fresh culture medium was added to the cells after 3 h, and the media was changed every 8 hours until the suspension cells were eliminated. Finally, MSCs were passaged and used for experiments after the third passage and before the 10^th^ passage (19). Meanwhile, we identified the MSCs. The osteogenic culture media and adipogenic culture media were added to MSCs, according to the manufacturer’s instructions (Cyagen, Shanghai, China). The differentiated MSCs were fixed in 4% paraformaldehyde and stained with Alizarin red and oil red O. Subsequently, the MSCs were collected, cleaned, and stained for flow cytometric analysis using anti-mouse CD11b-FITC, anti-mouse Sca-1-PE, anti-mouse CD45-PE, anti-mouse CD73-PE, anti-mouse CD90-PE and anti-mouse CD29-APC antibodies. The data were examined using the FlowJo program (TreeStar, Ashland, OR, USA).

### 2.4 Preparation of MSCs-derived exosome

To obtain the exosomes derived from MSCs, the MSCs were pre-cultured in a complete medium containing 10% exosome-free FBS for 48 hours to avoid contamination from the serum. The culture supernatants were collected for the isolation of exosomes, and the cells and debris were clarified by centrifugation: 300 g for 10 min, 2,000 g for 10 min, and 10,000 g for 30 min. Then, the exosomes were pelleted by ultracentrifugation of the supernatants at 100,000 g for 70 min and recovered by centrifugation in phosphate-buffered saline (PBS) (20). The exosomal proteins were estimated using the BCA test (Beyotime Biotech, Shanghai, China). The exosomes isolated from MSC supernatants were termed MEXs.

### 2.5 Identification of MEXs

Exosomal proteins (20µg) were separated on 12% SDS-PAGE and transferred to PVDF membranes. Then, the membrane was blocked with 5% non-fat dry milk at room temperature for 1 h, probed with primary antibodies to exosomal signature proteins (CD63, CD9, and TSG101) overnight at 4°C, and then incubated with horseradish peroxidase (HRP)-conjugated secondary antibodies (Beyotime Biotech, Shanghai, China) at room temperature for 1 h. Chemiluminescent reagents were used to visualize the immunoreactive bands on the membranes. GAPDH served as a loading control. MEXs were poured dropwise onto a 200-mesh carbon-coated copper mesh and set incubated at room temperature for 2 min, washed twice with PBS, and negatively stained with uranyl acetate for 5 min. The images of the exosomes were captured using a Philips CM12 transmission electron microscope at 80 kV. The qNano gold particle sizer was used to investigate the size distribution of MEXs.

### 2.6 Acute GVHD animal models

The mouse acute GVHD model was established according to a previously published protocol (19). Briefly, recipient (BALB/c) mice were fed water containing gentamicin sulphate (32×10^4^U/L) and erythromycin (250mg/L) 7 days before transplantation to prevent intestinal infection. On the day of transplantation, recipient mice received total body irradiation (TBI) with a dose of 8.0 Gy followed by tail intravenous injection of bone marrow cells (BMCs, 5×10^6^) and splenocytes (SCs, 1×10^7^) derived from donor C57BL/6 mice. At the same time, recipient mice were administered MEXs (300µg) and MSCs (3×10^5^) through tail vein injection according to the groups they were assigned to. The group in which the mice only received total body irradiation was termed the control group, the group in which the mice were injected with BMCs+SCs+PBS was termed the PBS group, the group injected with BMCs+SCs+MSCs was termed the MSC group, and the group injected with BMCs+SCs+MEXs was termed the MEX group. To examine the engraftment, BMCs of recipient mice were collected and the expression of MHC-I H-2k^b^, a gene-phenotype of C57BL/6 mice, was detected with flow cytometry at day 14 and day 28 after transplantation.

### 2.7 Effects of MEXs on dendritic cells

Dendritic cells (DCs) were induced as described in a previous study (21). Briefly, immature DCs (imDCs) were produced by culturing bone marrow mononuclear cells in the presence of IL-4 (10 ng/ml) and GM-CSF (20 ng/ml) for 6 days. To produce mature DCs (mDCs), the imDCs were further cultured with lipopolysaccharide (LPS, 1μg/ml) at day 6, and imDCs were harvested overnight. To investigate the effect of MEXs on the biological properties of DCs, MEXs (300 μg/ml) were added to the mDC culture, which was termed MEX-mDCs, or to the imDCs culture, which was termed MEX-imDCs, while the MSCs (MSCs: mDCs 1:10) were added to the mDC culture as a control, which was termed MSCs-mDCs. After 72 h, mDCs, imDCs, MSCs-mDCs, and MEXs-mDCs were collected and analyzed by flow cytometry. The supernatants were collected to measure the levels of IL-10, IL-6, and IL-12p70 by ELISA kits (Asbin, Shanghai, China).

### 2.8 Effects of MEXs on macrophages

In *in vitro* experiments, RAW 264.7 cells, a macrophage cell line, was stimulated with LPS (100 ng/ml) for 24 h. MSCs (MSCs: RAW264.7 1:1) and MEXs (300μg/ml) were added to the corresponding groups, respectively. After 48 h, RAW 264.7 cells were harvested. We, analyzed the expression of CD206, which is the main biomarker of M2 macrophages, by flow cytometry and real-time polymerase chain reaction (RT-PCR). In RT-PCR, total RNA was extracted by TRIzol (Takara), and then the Prime Script RT Master Mix kit (Takara) was used to reverse transcribe RNA into cDNA. The transcribed cDNA was employed as the template for real-time quantitative PCR on a Quant Studio™ 3 Real-Time PCR System (Applied Biosystems). Real-time PCR was conducted using the following gene-specific primers: *CD206* (forward): AGTCAGAACAGACTGCGTGG, *CD206* (reverse): CCAGAGGGAT- CGCCTGTTTT; *GAPDH* (forward): GGTTGTGTCCTGCGACTTCA, *GAPDH* (reverse): TGGTC -CAGGGTTTCT TACTCC. RT-PCR assay was performed in triplicate. The relative mRNA expression levels of CD206 were quantified using the 2^-ΔΔCT^ method and normalized using the housekeeping gene *GAPDH*.

In *in vivo* experiments, we isolated macrophages from the peritoneal lavage fluid of the mice by peritoneal lavage with RPMI-1640 medium at day 21 after transplantation, and the expression of CD206 in harvested cells was analyzed by flow cytometry.

### 2.9 Effects of MEXs on activation of lymphocytes

In *in vitro* experiments, mouse splenic CD4^+^ and CD8^+^ T lymphocytes were isolated using a magnetically labeled microbeads system (Miltenyi Biotec, Shanghai, China). The splenic CD4^+^ and CD8^+^ T lymphocytes were activated using mouse T activator CD3/CD28 beads (25 μl/ml (Invitrogen, Eugene, OR, USA). MSCs (MSC:T 1:10) and MEXs (300 μg/ml) were added to the corresponding groups, respectively. After 72 h, CD4^+^ and CD8^+^ T lymphocytes were collected and CD69 expression, which is a biomarker of active T cells, was assessed by flow cytometry.

In animal experiments, one mouse from each group was sacrificed on day 14 or day 21 after transplantation. At day 14, splenic cells were collected, and the ratio of Treg cells was examined by flow cytometry. At day 21, liver and lung tissues from the aforementioned mice were collected and then digested with collagenase 4 (Sigma, Shanghai, China); then, mononuclear cells were harvested and the proportion of CD4^+^ T cells was examined by flow cytometry.

To explore the effect of MEXs on the CD4^+^ and CD8^+^ T lymphocytes, splenic cells were collected and stained for flow cytometric analysis using anti-mouse CD4 APC, anti-mouse H-2kd-PE, and anti-mouse CD8-APC antibodies.

To explore the effects of MEXs on the distribution of Th1 cells and Th2 cells in transplanted mice, the levels of Th1 cell cytokines and Th2 cell cytokines in the plasma of recipient mice were examined by a cytometric bead array (CBA; BD Biosciences), following the manufacturer’s instructions. The analysis was performed using CBA software FCAP array™ v3.0.1.

### 2.10 Lymphocyte proliferation assay

To explore the effects of MEXs on lymphocyte proliferation, donor lymphocytes were labeled with CFSE and injected intravenously into recipient mice at a density of 1×10^7^ cells/mouse, and cell division and expansion were examined 5 days after cell injection. Splenic cells from recipient mice were collected and then CFSE-labeled lymphocytes were analyzed by flow cytometry.

### 2.11 Clinical and pathologic analysis of GVHD

The severity of GVHD was assessed every 3 days after transplantation based on five separate parameters, namely, weight loss, posture, activity, fur texture, and skin integrity, following the detailed description of the clinical GVHD scoring system, as described previously (22). Three weeks after transplantation, targeted organs (liver, lung, small intestine, and skin) from one mouse of each group were collected and the tissues from these targeted organs were fixed in 10% formalin, embedded in paraffin, stained with hematoxylin and eosin (H&E), and evaluated blindly for GVHD by a pathologist.

### 2.12 Cytotoxicity assay

To investigate the effect of MEXs on cytotoxicity against tumor cells of donor CD8^+^ T lymphocytes from recipient mice, we performed a cytotoxicity assay. Briefly, splenic CD8^+^ T lymphocytes from recipient BALB/c mice were collected as effector cells, and A20 cells, a leukemia cell line, were collected as targets with effector-target ratios ranging from 40:1 to 10:1. We used commercial cytotoxicity assays, based on lactate dehydrogenase (LDH) detection, following the manufacturer’s instructions (Cytotox 96, Promega, Shanghai, China). Meanwhile, the expression of perforin and Granzyme B of splenic CD8^+^ T lymphocytes from recipient BALB/c mice was also analyzed by flow cytometry.

### 2.13 Statistical analysis

Graphs were generated using the GraphPad Prism software (version 5.0.1; La Jolla, CA). Experiments were performed at least three times. Data were expressed as mean values ± standard deviation. One-way analysis of variance (ANOVA) was performed to assess the effects of a single factor on multiple groups. The animal sample size was calculated using sample size calculating software G*Power version 3.1.9.2 (Franz Faul, Universitaet Kiel, Germany) with an α-value of.05 and a power of 80%. All the P values were two-sided, and P < 0.05 was taken as statistically significant. The difference between the two groups was compared using an unpaired Student’s t-test. P<0.05 indicated a statistically significant difference.

## 3 Results

### 3.1 Characteristics of MSCs and MEXs

**Figure 1:**
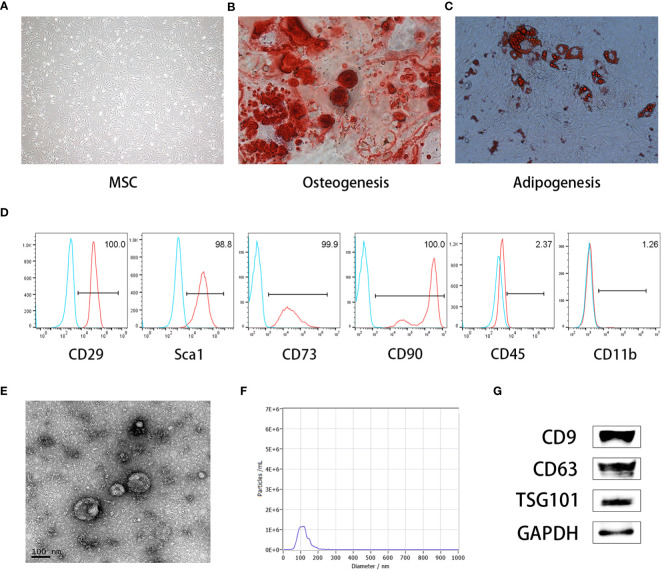
Characterization of MSCs and MSCs-derived exosomes (MEXs). **(A)**. Morphology of MSCs. **(B)**. MSCs were observed under an inverted microscope, MSCs can be differentiated into osteoblasts **(C)** and adipocytes **(D)**. **(E)**. Morphology of MEXs under electron microscopy. **(F)**. Particle size detection of MEXs. **(G)**. Exosomal signature protein of MEXs.

Under an inverted microscope, MSCs displayed adnate growth, homogenous cell distribution, and a polygonal, spindle-shaped, or star-shaped morphology, like fibroblasts (**Figure 1A**). The ability of MSCs to differentiate into different cell types was shown by discrete mineralized nodules and fat droplets after osteogenic and adipogenic stimulation and alizarin red and oil red O staining (**Figures 1B, C**). Immunophenotyping showed that more than 95% of the cells exhibited CD29 (+), Sca1 (+), CD70 (+), CD90 (+), CD45 (–), and CD11b(-) phenotypes, which presented typical immunophenotypes of MSCs (**Figure 1D**). Meanwhile, we characterized the MEXs derived from MSCs using electron microscopy and nanoparticle tracking analysis (NTA). Our data showed that the MEXs were physically homogeneous, exhibited the dimpled, cup-shaped characteristic morphology, and were of a size range of 40 to 160 nm in diameter (**Figures 1E, F**). Furthermore, western blot analysis indicated that the MEXs expressed CD9, CD63, and TSG101, which are considered typical exosomal proteins, and were negative for the endoplasmic reticulum protein GRP94 (data not showed) (**Figure 1G**).

### 3.2 MEXs attenuate dendritic cell maturation and function

**Figure 2:**
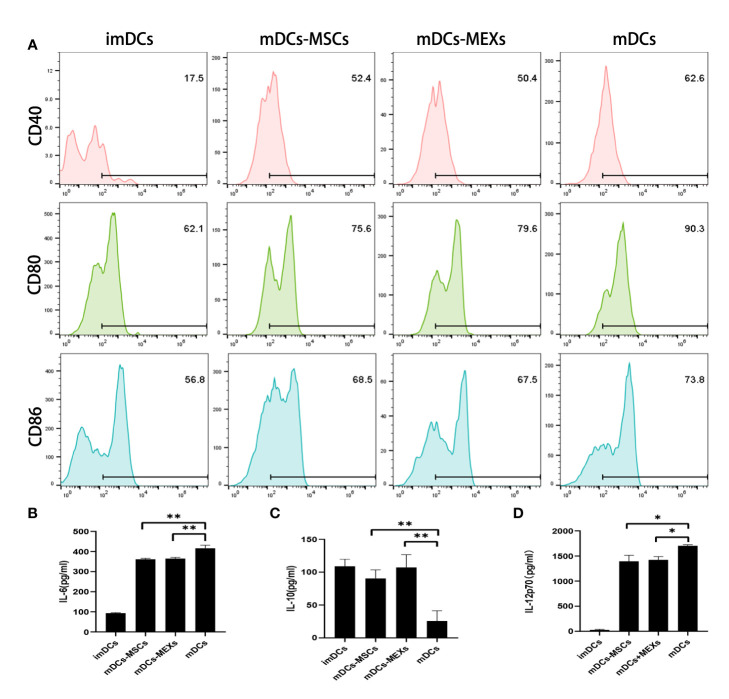
Effect of MEXs on the maturation and activation of DCs. LPS (1ug/ml)-induced activation of imDCs to mDCs, and addition of MEXs or MSCs to mDCs culture, which were termed MEXs-mDCs and MSCs-mDCs, respectively. **(A)**. CD40, CD80, and CD86 expression were detected on CD11c+ DCs. The levels of IL-6 **(B)**, IL-10 **(C)**, and IL-12p70 **(D)** in the supernatant were measured by ELISA. Data are representative of three independent experiments and are presented as the mean ± SD. *P < 0.05, **P < 0.01.

To investigate the effect of MEXs on the biological properties of dendritic cells (DCs), we evaluated the effects of MSCs on the expression of co-stimulatory molecules and functional cytokines secretion of DCs. Co-stimulatory molecules CD80/CD86 attach to CD28 to provide a costimulatory signal for T lymphocyte activation. IL-6 and IL-12 are pro-inflammatory cytokines, whereas IL-10 is an anti-inflammatory cytokine. As shown in **Figure 2**, the expression of CD40, CD80, and CD86 on mDCs was significantly downregulated after treatment of MEXs or MSCs compared to mDCs without treatment of MEXs or MSCs (**Figure 2A**), indicating that, like MSCs, MEXs can also suppress the maturation of dendritic cells. Meanwhile, the levels of IL-6 and IL-12p70 in the supernatants of mDCs treated with MEXs and MSCs were notably lower than those in the mDCs group without treatment of MEXs and MSCs. However, IL-10 was markedly upregulated in the MEXs and MSCs treated group. Taken together, our data suggested that, like MSCs, MEXs can attenuate the maturation and immune function of DCs.

### 3.3 Engraftment

**Figure 3:**
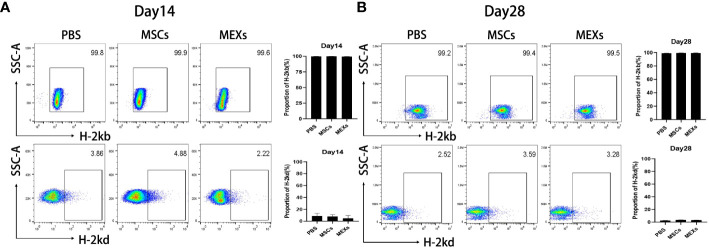
Analysis of engraftment. MSCs or MEXs were injected into recipient mice three times within 1 week after transplantation. The proportion of H-2k^b^positive cells and the proportion of H-2k^d^positive cells in the bone marrow of Babl/c recipient mice at day 14 **(A)** and day 28 **(B)** after transplantation were examined by flow cytometry. Data are representative of three independent experiments and are presented as the mean ± SD.

To confirm the engraftment of donor cells in recipient mice and whether administration of MEXs and MSCs affects the engraftment of donor cells, we examined the expression of H-2k^b^, a donor genotype at day 14 or day 28 after transplantation. As shown in **Figure 3**, over 95% of the bone marrow cells showed the donor genotype (H-2k^b^) in all groups, indicating that the donor cells were implanted successfully and that MEXs and MSCs did not affect the engraftment of donor cells.

### 3.4 MEXs can promote M2-type macrophage polarization

**Figure 4:**
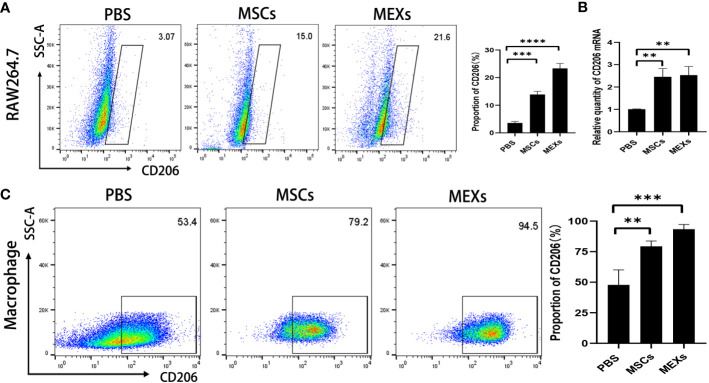
Effects of MEXs on CD206 expression of RAW 264.7 cell line and mice peritoneal macrophages. After 3 days of co-culturing with MSCs or MEXs, CD206 expression of RAW 264.7 cells was detected by flow cytometry **(A)** and real-time PCR **(B)**. **(C)** CD206 expression of mice peritoneal macrophages at day 21 after transplantation. Data are representative of three independent experiments and are presented as the mean ± SD. **P < 0.01, ***P < 0.001.

Macrophages are divided into classically activated M1 type (pro-inflammatory) and selectively activated M2 type (anti-inflammatory). These two major macrophage subpopulations exert different functions, including classically activated/inflammatory (M1) and alternatively activated/regenerative (M2) macrophages, and show different phenotypes. Flow cytometry was used to detect the proportion of CD11b^+^CD206^+^ M2-like macrophages and CD11b^+^iNOS^+^ M1-like macrophages in total macrophages (CD11b^+^F4/80^+^ macrophage). In this study, we first treated RAW 264.7 cell line, a mouse macrophage cell line with MEXs and MSCs, to explore the effect of MEXs on the phenotype of macrophages *in vitro.* As shown in **Figure 4**, the expression of CD206 was notably higher in the MEXs-treated group compared to the PBS control group and was similar to that in the MSCs-treated groups (**Figure 4A**, p<0.001 and 0.01). To confirm this data, we also examined the CD206 mRNA expression of RAW 264.7 cells treated with MEXs. Our data showed that, like MSCs, MEXs also significantly elevated the CD206 mRNA expression of RAW 264.7 cells (**Figure 4B**), indicating that MEXs can promote the conversion of M1 to M2-type macrophages *in vitro*. Whether MEXs could promote macrophage polarization toward M2 *in vivo* is unclear. The proportion of macrophages expressing CD206 in the peritoneal macrophages from the mice in the MEXs or MSCs groups was examined. As shown in **Figure 4C**, like the MSCs-treated group (79.2%), the proportion of macrophages expressing CD206 in the MEXs group (94.5%) was markedly higher than that in the PBS group (53.4%, p<0.001), indicating that MEXs can also induce macrophage polarization toward M2 type *in vivo*. Taken together, our data suggest that MEXs can induce macrophage polarization toward M2 type *in vitro* and *in vivo*, which may be one of the potential mechanisms by which MEXs can alleviate GVHD.

### 3.5 MEXs can exert immunosuppressive capacity on T lymphocytes

**Figure 5:**
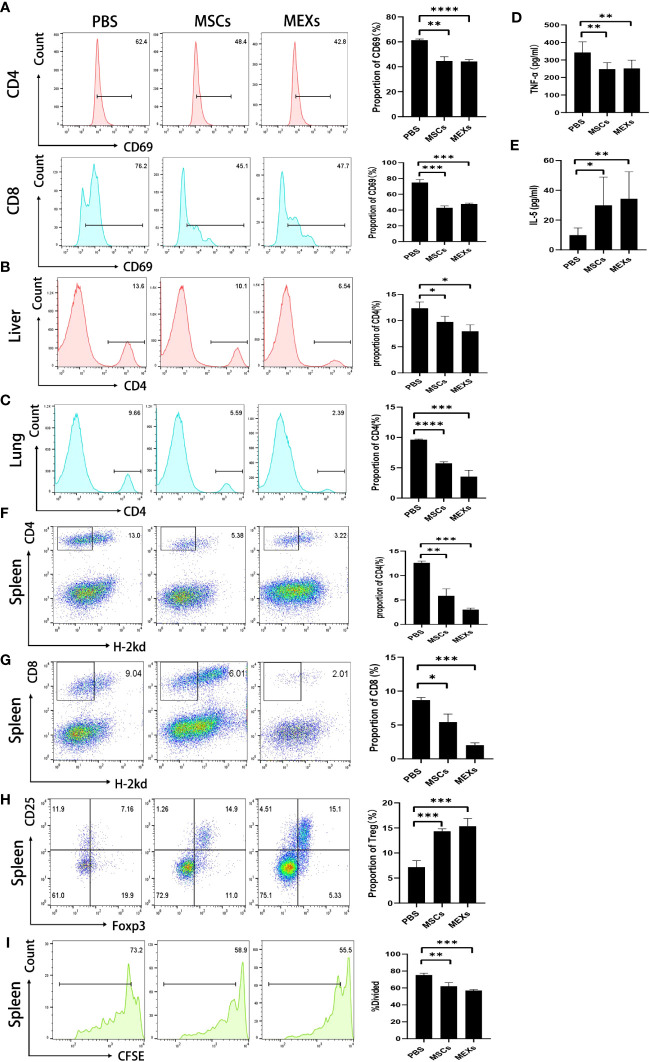
Effects of MEXs on activation and proliferation of lymphocytes. **(A)** After 3 days of co-culturing with MSCs and MEXs, CD69 expression of CD4^+^T cell or CD8^+^T cell was analyzed using flow cytometry. On day 21 after transplantation, the proportion of CD4^+^T cells in mononuclear cells from the liver **(B)** and lungs **(C)** was detected by flow cytometry. The levels of TNF-α **(D)** and IL-5 **(E)** in the serum of transplanted mice were detected by CBA assays. **(F)** The proportion of CD4^+^H-2kd^-^ cells was detected by flow cytometry on day 14 after transplantation. The levels of TNF-α**(D)** and IL-5**(E)** in the serum of transplanted mice were detected by CBA assays. **(G)** The proportion of CD8^+^H-2kd^-^ cells was detected by flow cytometry on day 14 after transplantation. **(H)** The proportion of CD4^+^CD25^+^Foxp3^+^ Treg cells was detected by flow cytometry on day 14 after transplantation. **(I)** Proliferation of donor splenic cells analyzed by CFSE assays at day 5 after transplantation. Data are representative of three independent experiments and are presented as the mean ± SD. *P < 0.05, **P < 0.01, ***P < 0.001, ****<0.0001.

To investigate whether MEXs could exert a suppressive effect on the function of T lymphocytes, we first isolated lymphocytes from the spleen of C57BL/6 mice, and then cocultured them with MEXs and MSCs *in vitro*, respectively. The coculture system was supplemented with mouse T cell activator CD3/CD28 beads. After 72 h, CD4^+^ and CD8^+^ T lymphocytes were harvested and CD69 expression was assessed by flow cytometry. As shown in **Figure 5A**, the proportion of CD4^+^ and CD8^+^ T lymphocytes expressing CD69 in groups cultured with MEXs and MSCs was significantly lower than that in the PBS group (p<0.001), indicating that, like MSCs, MEXs can exert a suppressive effect on T lymphocyte activation *in vitro*. Meanwhile, the effects of MEXs on the infiltration of lymphocytes in organs were examined *in vivo*. The mice were injected with MEXs and MSCs, and the mononuclear cells derived from tissues of the livers, lungs, which are usually regarded as target organs for GVHD, and spleens were harvested, and the proportion of CD4^+^ T lymphocytes was examined at day 21. The proportion of CD4^+^ T lymphocytes in liver (**Figure 5B**) and lung (**Figure 5C**) tissues from the mice in MSCs and MEXs groups was markedly less than that in the PBS control groups (p<0.05 and p<0.001), indicating that MEXs can notably inhibit the infiltration and immigration of T lymphocytes to target organs *in vivo*.

To investigate whether MEXs could exert a suppressive effect on the function of T lymphocytes *in vivo*, splenic cells were collected and stained for flow cytometric analysis. As shown in **Figures 5F, G**, the proportion of donors’ CD4^+^ and CD8^+^ T lymphocytes in groups cultured with MEXs and MSCs was significantly lower than that in the PBS group, which was consistent with the results *in vitro*.

To investigate the effect of MEXs on T helper cells *in vivo*, a mouse Th1/Th2 cytokine kit was used to measure the levels of Th1 cytokine TNF-α and Th2 cytokine IL-5 in the serum of previously transplanted mice. Compared to the PBS control group, the lower levels of TNF-α and the higher levels of IL-5 were observed in mice treated with MEXs (**Figures 5D, E**). These results were similar to that in the MSCs group, indicating that, like MSCs, MEXs can promote Th1 to Th2 cytokines shift, which may contribute to alleviating the onset of acute GVHD in allo-HSCT. Meanwhile, we also examined the effect of MEXs on the differentiation of regulatory T cells (Treg cells). At day 14, the proportion of CD4^+^CD25^+^Foxp3^+^ Treg Cells in spleens was detected by flow cytometry. As shown in **Figure 5H**, the proportion of CD4^+^CD25^+^Foxp3^+^ Treg cells was significantly higher in the MEXs (p<0.001) and MSCs groups (p<0.001) than in the PBS control group, indicating that, like MSCs, MEXs can also exert a critical role in encouraging T lymphocytes to differentiate into Treg cells, which play key roles in suppressing autoimmunity and in maintaining immune homeostasis (23, 24).

In the T lymphocyte proliferation assay, donor splenic cells were labeled with CFSE and injected intravenously into irradiated recipients at a density of 1×10^7^ cells/mouse, and cell division and expansion were examined 5 days after cell injection. The proliferation of T lymphocytes in spleens was analyzed by visualizing CFSE fluorescence with a flow cytometer. As shown in **Figure 5I**, like MSCs, MEXs can notably suppress the proliferation of activated T lymphocytes in spleens compared with the control group; the proliferation rates for the groups of MSCs, MEXs, and PBS were 58.65 ± 3.45, 55.38 ± 1.05, and 72.97 ± 1.63, respectively. This occurrence was notable in the MEXs group, indicating that MEXs can inhibit lymphocyte proliferation *in vivo*. Taken together, our data suggest that, like the parental MSCs, MEXs can exert immunosuppressive activity on T lymphocytes.

### 3.6 MEXs can alleviate the severity of acute GVHD and improve overall survival in a murine model

Whether the administration of MEXs can alleviate the severity of acute GVHD or not is under debate since MEXs can exert immunosuppressive activity on DCs and T lymphocytes. We performed acute GVHD clinical scores in each group of mice every 4 days from day 1 to day 29 after transplant.

**Figure 6:**
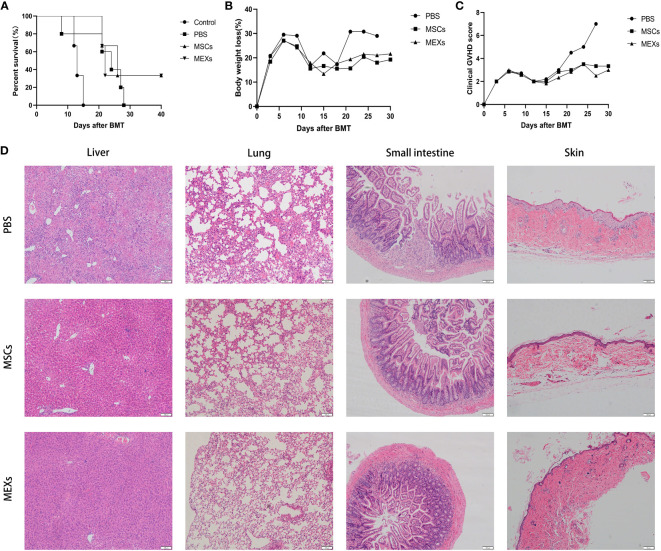
Effects of MEXs on the occurrence of GVHD. **(A)** Overall survival of mice in each group. **(B)** Body weight loss of mice in each group. **(C)** Clinical GVHD score of mice in each group. **(D)** Representative histopathology images of the liver, lungs, small intestine, and skin tissue on day 21 after transplantation. Data are representative of at least three independent experiments for A-D.

The mice that received only TBI without BMC infusion began to die on day 13 after transplantation, and all died within 16 days. More than 90% of mice in the BMCs + SCs +PBS group, as a control group, gradually died within 28 days after transplantation, whereas more than 20% of mice in the MSCs and MEXs groups were still alive at the end of the 40-day observation period (**Figure 6A**). The mice in the control group exhibited significant weight loss compared with the mice in the MSCs and MEXs groups (**Figure 6B**, p<0.05). Meanwhile, the GVHD clinical score in the MSCs and MEXs groups was markedly lower than that in the control group (**Figure 6C**, p<0.01). To confirm this outcome, we also performed a histopathology examination. As shown in **Figure 6D**, our data displayed hepatocyte edema and necrosis, massive inflammatory cell infiltration in the confluent area, and disorganized liver lobules in the liver of mice from the control group, consistent with GVHD. However, these histopathology characteristics of acute GVHD in livers from the mice in the MSCs and MEXs groups were significantly reduced. The alveolar septum was thick and perivascular inflammatory cells surrounded multiple layers in the lung of mice from the control group, but these histopathology characteristics of acute GVHD in the lung of mice in MSCs and MEXs groups were notably reduced. Most of the mice in the control group showed diarrhea, and histopathology displayed a destroyed small intestinal villi structure, inflammatory cell infiltration in the submucosal layer, and abscess formation in the crypt, while these histopathology characteristics of acute GVHD in the small intestine of mice in the MSCs and MEXs groups were significantly alleviated. Skin is the most common target organ for GVHD, and the skin histopathology of mice in the control group showed severe epidermal hyperplasia, thickened spiny layer, and incomplete keratinization, while the skin histopathology of mice in the MSCs and MEXs groups showed a normal skin structure. Taken together, our data suggest that MEXs can alleviate the severity of acute GVHD and improve overall survival in a murine model.

### 3.7 MEXs can preserve antitumor CTL activity

**Figure 7:**
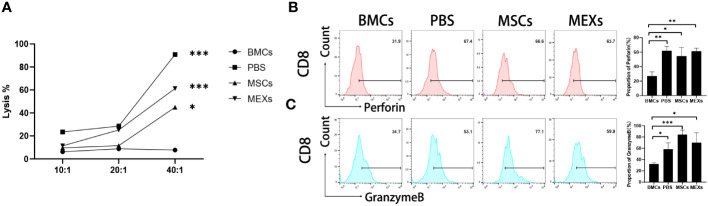
Cytotoxicity assay. **(A)** The cytotoxic activity against A20 cells of CD8^+^ T cells from recipient mice treated with MSCs or MEXs. The expression of Perforin **(B)** and Granzyme B **(C)** of CD8^+^ T cells from recipient mice treated with MSCs or MEXs. Data are representative of three independent experiments for **(A–C)**. Data are presented as the mean ± SD. *P < 0.05, **P < 0.01, ***P < 0.001.

Whether MEXs can affect the CTL activity of donor T lymphocytes is under debate, since MEXs can inhibit lymphocyte proliferation *in vivo*. The anti-leukemia cytotoxic T lymphocyte (CTL) activity of T cells derived from recipients post-transplantation was assessed. At day 14, the splenic CD8^+^ T lymphocytes from recipient mice were collected as effector cells, and the A20 leukemia cell line was used as a target. As shown in **Figure 7A**, the cytotoxicity of T cells against the A20 cells from mice in the BMCs+ SCs+ PBS (PBS group), BMCs+ SCs+ MSCs (MSCs group), and BMCs + SCs + MEXs (MEXs group) groups were stronger than that of the BMCs group at the effect target ratio of 20:1; these differences were more pronounced at the effect target ratio of 40:1(p<0.001) and no obvious differences were observed between the MSCs or MEXs groups and PBS group. Meanwhile, our data also revealed that the expression of Perforin (**Figure 7B**) and Granzyme B (**Figure 7C**) in splenic CD8^+^ T lymphocytes from the mice in the MSCs group and MEXs group were notably higher than that in the BMCs group, and no differences were observed among the other three groups, indicating that administration of MSCs and MEXs could not affect the anti-leukemia activity of CD8^+^ T lymphocytes from recipients. In conclusion, our data suggest that although, like MSCs, MEX cells could inhibit T cell proliferation and alleviate the severity of acute GVHD, the antitumor CTL activity was preserved.

## 4 Discussion

One of the main factors contributing to the failure of allogeneic HSCT is acute GVHD, which is one of the most important causes of morbi-mortality after allo-HSCT. Classically, three stages are involved in the development of acute GVHD (25). First, tissue damage from the conditioning regimen mediates the activation of APCs. Second, donor T lymphocytes are activated by recipient antigens presented by host APCs. Third, donor T lymphocytes attack target tissues and cause damage. The first phase primarily relates to tissue damage, such as liver and intestine damage from radiotherapy pretreatment prior to transplantation. This promotes the secretion of pro-inflammatory cytokines and growth factors, such as TNF-α, IFN-γ, IL-1, and GM-CSF, which induces adhesion and expression of MHC molecules and promotes the recognition of allogeneic antigens via donor T lymphocytes that uptake the antigen and multiply for activation in the second stage. Duffner et al. used APC (II^+/+^) expressing MHC II-deficient (II^-/-^) mice to examine the capacity of DCs and B cells to initiate acute GVHD (resistant to CD4-dependent GVHD). Consequently, injecting host-derived II^+/+^ DCs or host-derived II^+/+^ B cells alone was sufficient to produce deadly acute GVHD in II^-/-^ animals and break down GVHD resistance. In contrast, donor CD4^+^ T lymphocytes could not be activated or tolerated by primitive or LPS-stimulated host-derived II^+/+^ B cells. These findings implied that donor CD4^+^ and CD8^+^ T lymphocytes must first be activated by the host-derived DCs to trigger GVHD (25). Additionally, decreasing the T lymphocytes in donor cells would improve the symptoms of GVHD and alleviate the severity of GVHD. However, donor versus recipient immune reactions also harbored a beneficial effect since they mediated the immunological eradication of residual tumor cells, in the context of the so-called GVL effect, which could eradicate the residual leukemia cells. Therefore, drastically lowering the amount of T lymphocytes would weaken the GVL effect. The third phase is the effector phase, in which various immune cells exert tissue killing against the targeted organs of recipients that do not match the donor MHC type under the regulation of inflammatory factors.

Currently, the prevention of acute GVHD is achieved using calcium phosphatase inhibitors, namely, methotrexate, which suppresses donor T lymphocytes in the graft and may also weaken the anti-leukemic effect of the graft, which would increase the relapse and graft failure and thus decrease the survival rate of the patient. Therefore, prevention and treatment of acute GVHD while preserving GVT is the research hotspot in allo-HSCT.

MSCs have a wide spectrum of immunomodulatory characteristics in addition to stem/histocyte traits. In a non-inflammatory setting (low levels of TNF-α and IFN-γ), bone marrow MSCs adopt a pro-inflammatory phenotype and enhance T lymphocyte response by secreting chemokines to recruit lymphocytes for infection. In an inflammatory setting, MSCs are activated and transformed into an immunosuppressive phenotype, secreting high levels of soluble factors, such as IDO, PGE2, NO, TGF-β1, IL-10, and hepatocyte growth factor. Moreover, MSCs can suppress T lymphocyte activation, prevent Th1 and Th17 cell differentiation, promote macrophage polarization toward M2 for anti-inflammatory effects, and inhibit dendritic cell activation and maturation (26). Depending on their immune properties, MSCs can be used to treat a wide range of disorders, including osteoarthritis, cirrhosis from hepatitis, pulmonary fibrosis, inflammatory bowel disease, and other autoimmune diseases. Recent studies have shown that MSC-based treatment promises great results in acute GVHD, with several clinical trials demonstrating the efficacy of MSCs in steroid-refractory acute GVHD (27, 28).

As early as the 1960s, it was observed that there were membrane vesicles in the extracellular space, which were termed extracellular vesicles (EVs). EVs contain membrane molecules, cytoplasmic proteins, and nucleic acid molecules such as DNA and RNA from mother cells. The biological characteristics of EVs are closely related to the proteins and RNAs they contain. EVs participate in important physiological and pathological processes in the body, including antigen presentation, immune response, angiogenesis, and inflammatory response. Exosomes are the main component of EVs, and studies on MSC-EVs mainly refer to MSCs-derived exosomes (MEXs) (29–32). Recent studies have shown that MSC-EVs and MEXs have similar functions to their parental MSCs and even broader clinical application potential (15, 32, 33). However, the effects of MEXs on immunomodulation and the application potential in acute GVHD are unclear.

In the present study, we first characterized the MEXs using electron microscopy and NTA. Our data showed that, like other cell exosomes, the MEXs were also dimpled, with a cup-shaped morphology with size of 40 to 160 nm in diameter, and expressed HSP70, CD9, CD63, and TSG101, as well as were negative for the endoplasmic reticulum protein GRP94. In terms of immunomodulation, studies have shown that MSC-EVs can inhibit the activation of T cells and dendritic cells, inhibit the development and differentiation of Th1 and Th17 cells, and induce immune cells to secrete IL-10 and TGF- β immunosuppressive factors, which can inhibit lymphocyte proliferation and induce Treg cell generation (14, 15, 34). As the main component of MSC-EVs, MEXs can exert the immunomodulation function like their parental MSCs. Next, we examined the effects of MEXs on the function of immune cells, such as dendritic cells, T lymphocytes, and macrophages *in vitro* and found that, like MSCs, MEXs not only inhibited the expression of CD80 and CD86 on dendritic cells (DCs) but also inhibited their functional cytokines IL-6 and IL-12p70 secretion, indicating that, like their parent MSCs, MEXs can also suppress the immune activity of DCs *in vitro*. Studies have shown that microRNAs were significantly enriched in MSC-EVs compared to their parent MSCs. MicroRNAs known to have an impact on DC maturation and function include miR-21-5p, miR-142-3p, miR-223-3p, and miR-126-3p (15). MSC-EVs can recapitulate MSC-mediated DC modulation, and microRNAs enclosed with MSC-EVs may represent a novel mechanism through which MSCs modulate DC functions. Meanwhile, another study showed that, as the main component of MSC-EVs, MSCs‐derived exosomes (MEXs) can also decrease the surface marker expression in DCs treated with LPS, decrease IL‐6 release, whereas it can augment IL‐10 and TGF‐β1 release and decrease lymphocyte proliferation in the presence of DCs treated with MEXs (15). Our data are consistent with these studies. Taken together, these findings suggest that, like parental MSCs, MEXs can suppress the function of DCs, indicating that they may be important modulators of DC‐induced immune responses.

Donor T lymphocytes play an important role in the onset of acute GVHD, and because MSCs can suppress T lymphocyte activation, MSCs can be used to treat acute GVHD. In this study, we found that MEXs can significantly decrease CD69 expression of CD4+ and CD8^+^ T lymphocytes, indicating that, like MSCs, MEXs can also exert activation-inhibitory effects on T lymphocytes *in vitro*. Moreover, we also found that, like MSCs, MEXs can markedly suppress the proliferation of T lymphocytes in the spleen of mice allo-HSCT models. Taken together, our data showed that MEXs can exert suppressive effects on the activation and proliferation of T lymphocytes. Th1/Th2 cells play a critical role in the pathogenesis of GVHD. Some studies have shown that the cytokine profile of effector cells induces predominantly GVL effects with reducing GVHD across MHC and minor histocompatibility antigen barriers, which may be related to a Th1 to Th2 cytokine shift (35–38). Donor Th1 cells preferentially secrete type-1 cytokines (IL-2, IFN-γ, and TNF-α) and induce GVHD, whereas donor Th2 cells, which secrete type-2 cytokines (IL-4, IL-5, IL-10, and IL-13), could reduce GVHD (39–41). Th1 cytokines exert immune activation, enhance the activation and recruitment of macrophages and B cells, and promote cytotoxic T-cell proliferation to induce acute GVHD. However, Th2 cytokines are associated with immunologic tolerance and reduce the production of causative agents of acute GVHD (42). To investigate whether this shift was maintained in recipients after administration of MEXs in an allo-HSCT animal model, we further analyzed the Th1 cytokines TNF-α and Th2 cytokines IL-5 production of splenocytes from recipients 14 days after transplantation. We found that there was a significant increase in IL-5 production, but a significant decrease in TNF-α production in the mice treated with MSCs and MEXs compared with that in the control group, indicating that administeringMSCs and MEXs following allo-HSCT still resulted in a Th1 to Th2 cytokine shift.

Moreover, we also found that, like MSCs, MEX-treated mice showed an increase in Treg cells as compared to the control group. Previous studies in allo-HSCT animal models showed that the addition of highly purified CD4^+^CD25^+^FoxP3^+^Treg cells resulted in a reduction of acute GVHD (43). We hereby speculate that increased Treg cells might be involved in regulating the immune response in acute GVHD mice treated with MEXs; these studies further indicate the therapeutic potential of MEXs in GVHD.

Macrophages exhibit versatility in phenotype and function (44). The M1-like macrophages mediate pro-inflammatory effects with upregulated expression of inducible nitric oxide (iNOS), while the M2-like macrophages exert immunosuppressive effects with expression of CD206 and arginase 1 (Arg1) (45). Previous studies demonstrated that M1-like macrophage plays an important role in the pathogenesis of acute GVHD (46), and human skin lesions infiltrated by macrophage were proposed as a predictive factor for refractory GVHD associated with poor overall survival rate (47). On the contrary, M2-like macrophages can reduce the inflammatory response (48). Therefore, different subsets of macrophages play different roles in the inflammation process, while GVHD is caused by donor T cell–mediated damage to recipient target organs either via cytolytic attack or release of inflammatory mediators. Ours and other studies indicated that macrophages derived from mice treated with MSCs showed high expression of the M2 marker CD206, and a reduced expression of M1 marker iNOS, indicating that MSCs can promote M2 macrophage polarization (19, 49, 50). In line with our findings, miR-216a-5p was abundant and strongly expressed in hypoxia-treated MEXs, and possibly engaged in MEX-mediated microglia to M2 polarization (51).Taken together, our data suggest that, like parent MSCs, MEXs can induce M2 macrophage polarization *in vitro* and *in vivo*. MEXs can shift macrophages to M2-like subsets, and MEXs may have a therapeutic potential for GVHD.

MSC-EVs have been used in several preclinical studies due to their immunomodulatory properties. Seo et al. reviewed the immunomodulatory effects of MSC-EVs in animal models of GVHD, inflammatory bowel disease, sepsis, and type 1 diabetes mellitus, and found that MSC-EVs may be useful in the treatment of inflammatory diseases through direct immunosuppressive functions to alleviate the symptoms of immune disorders or to improve the xenotransplantation efficiency (52). Similarly, MSC-EVs have great therapeutic potential in acute graft-versus-host disease, and as early as 2014, Kordelas et al. reported that GVHD symptoms were severely suppressed for more than 4 months after treating patients with allogeneic MSC-EV products (53). Gupta et al. conducted a review of preclinical studies of MSC-EVs for the treatment or prevention of graft-versus-host disease and showed that the use of MSC-EVs in the treatment and prevention of GVHD improved overall survival rate and GVHD clinical scores and attenuated GVHD-induced inflammatory damage (54), indicating that MSC-EVs are promising in the treatment and prevention of GVHD. In the present study, we showed that there was a lower incidence of GVHD and a higher rate of leukemia-free survival in mice receiving MSC and MEX administration. The severity of GVHD was lessened, the mice’s body weight loss was decreased significantly, and the survival rate was increased in the MEXs group compared to that in the control group. Meanwhile, we also examined the antitumor response of T lymphocytes derived from recipients and found that T cells obtained from recipient mice in the MEXs group exhibited a stronger antitumor response than that in the PBS control group. Moreover, the perforin expression of effector T lymphocytes was the same as that in the PBS group and significantly higher than that of the BMCs group, while the level of granzyme expression of effector T lymphocytes was similar to that in the PBS group. Wu et al. demonstrated that miR-17-92 boosts CD8^+^ T lymphocyte migration to GVHD target organs but has little influence on CD8^+^ T lymphocyte proliferation, survival, or cytolytic capability, which might retain the benefits of MEXs on GVL (37). Taken together, our results suggest that, although specific T cell tolerance was induced, the antitumor CTL immune response was still maintained in our transplant model.

Intravenous delivery of MSCs has gradually entered clinical trials for therapeutic and immunomodulatory use in various tissue regeneration trials. However, studies have shown that most administered MSCs are blocked by the pulmonary barrier, while MEXs avoid this disadvantage because of their small size (55). This phenomenon was also confirmed in our experiments, in which the proportion of CD4^+^ T lymphocytes in the lungs was lower after MEX treatment than after MSC treatment. Exosomes can be stored at −20°C to 37°C. However, exosomes underwent minor structural changes at the conditions of 4°C and 37°C (18), but exosomes maintained at −20°C were stable and did not undergo repeated freezing and thawing, making them simple to store and transport at 4°C and 37°C (18). MSCs can be cultured once to yield a significant volume of cell supernatant, and the separated exosomes, frozen and stored to guarantee uniform quality and reliable outcomes, were utilized. This avoids the labor-intensive process of culture, harvesting, and purification required for each MSC used, which increases the hazard of contamination by infectious pathogens, such as mycoplasma, viruses, endotoxins, or prions (18). Over the period of 10 generations, we discovered that MEXs produced from MSCs lost most of their immunomodulatory activity, which might be connected to the aging of their parental cells (56).

While many animal disease models and clinical trials have suggested the efficacy of MSCs for the treatment of graft-versus-host disease and immune system disorders, a number of clinical results disprove this (57). A randomized, placebo-controlled phase III trial evaluating MSC infusion as a steroid-refractory treatment demonstrated that the addition of MSCs produced significant improvement without additional toxicity in patients with GVHD involving visceral organs, particularly the liver and intestines, but failed to meet the primary endpoint. There may be a number of reasons for these differences, including the source of MSCs, the culture method, the dose and mode of administration, and the evaluation criteria, but the most likely reason is that different stages of the disease and changes in the *in situ* inflammatory state of the recipients may differentially affect the activation state of the MSC (57–59).

Exploiting the plasticity of MSCs to different microenvironments could optimize the therapeutic efficacy of MSCs. Mendt et al. used pro-inflammatory cytokines to metabolically reprogram MSCs to increase their immunosuppressive potential and improve outcomes in a heterozygous mouse model of GVHD (60). Stimuli received by MSCs from the environment also affected EV content and their effects on target cells, potentially enhancing their immunomodulatory and regenerative properties, for example, hypoxic conditions can activate and modulate signaling pathways in MSCs, enriching the EV content of specific molecules, thereby enhancing their tissue repair capacity and immunomodulatory properties (54, 61, 62).

The quality control of MSCs is the main challenge for its clinical application. It is difficult to perform quality control of the culture process, and the storage and transportation conditions can significantly affect the cellular efficacy, which leads to the limitation of the clinical application of MSCs (63). Due to the small size, high stability, and cell-free characteristics of MSC-EVs, they are easy to store and transport for a long period of time, which makes the application of MSC-EVs more convenient, less costly, safer, and more effective from the perspective of clinical utility (18, 64). In addition, studies have also shown that EVs secreted by different cells carry the biological characteristics of their membrane cells, which can cross the blood-brain barrier more easily than cells, and can be used for judging the efficacy of various diseases or as a drug delivery system (65, 66). Although MSC-EVs are a promising immunomodulatory therapy, there are still some challenges in the clinical application of MSC-EVs. MSC-EVs are derived from MSCs, and every part of the MSC culture process may affect the quantity and quality of EVs, in addition, there is no recognized efficient EV isolation method and quality control. How to obtain high purity large-scale clinical application grade of MSC-EVs is still the main problem at present (52).

In conclusion, the data implied that MEXs had similar immunomodulatory capabilities as their parent MSCs and could exert a GVHD therapeutic impact while maintaining the GVL effect in allo-HSCT, although some of these processes are yet unclear. We need to further explore the maximum benefits of MEXs.

## Data availability statement

The raw data supporting the conclusions of this article will be made available by the authors, without undue reservation.

## Ethics statement

The animal study was approved by the Ethics Committee of Xinhua Hospital Affiliated to the Shanghai Jiao Tong University School of Medicine. The study was conducted in accordance with the local legislation and institutional requirements.

## Author contributions

YJ: Formal Analysis, Methodology, Writing – original draft, Writing – review & editing. JZ: Data curation, Formal Analysis, Methodology, Writing – original draft. MW: Data curation, Formal Analysis, Methodology, Software, Writing – original draft. FH: Data curation, Formal Analysis, Methodology, Software, Writing – review & editing. JL: Data curation, Methodology, Software, Writing – review & editing. RL: Data curation, Methodology, Software, Writing – review & editing. JW: Investigation, Methodology, Writing – review & editing. SH: Funding acquisition, Investigation, Methodology, Project administration, Writing – review & editing, Writing – original draft.
